# Using a model to design, implement, and evaluate a training program for improving cultural competence among undergraduate nursing students: a mixed methods study

**DOI:** 10.1186/s12912-022-00849-7

**Published:** 2022-04-11

**Authors:** Jamileh Farokhzadian, Monirsadat Nematollahi, Nahid Dehghan Nayeri, Motahareh Faramarzpour

**Affiliations:** 1 Nursing research center, Kerman University of medical science, Kerman, Iran; 2 Nursing and Midwifery Care Research Centre, School of nursing and Midwifery, Tehran University of Medical Sciences, Tehran, Iran; 3 Student Research Committee, Razi faculty of Nursing and Midwifery, Kerman University of Medical Sciences, Kerman, Iran; 4 Clinical Research Development Center of Imam Khomeini Hospital, Jiroft University of Medical Sciences, Jiroft, Iran

**Keywords:** Instructional design, Cross-cultural care, Culturally competent, Culturally competent care, Culturally congruent care, Multicultural care, Transcultural nursing

## Abstract

**Background:**

Due to changing population, culturally diverse clients with different perceptions of illness and health are present in healthcare settings. Therefore, it is increasingly important for nursing students to have high levels of cultural competence in order to meet diverse client needs. A training program is essential to enhance students’ cultural competence. This study aimed to design, implement, and evaluate a cultural care-training program to improve cultural competence of undergraduate nursing students.

**Methods:**

This exploratory mixed methods study used six steps proposed in the Talbot and Verrinder model to design a training program. In the first step, a conventional qualitative study was conducted and 18 participants were interviewed using purposive sampling. In the second and third steps, literature review and the classic Delphi technique were used for initiation and finalization of the program. The fourth, fifth, and sixth steps were completed by implementing, monitoring, and evaluating the cultural care program (five two-hour sessions) among 73 nursing students using a quasi-experimental design. Finally, effectiveness of program was evaluated through the cultural care inventory before and 1 month after the program. Data were analyzed via SPSS25, independent samples t- test, paired t- test, chi-square test, analysis of covariance, and multivariate linear regression tests.

**Results:**

A systematic model was used to identify key elements of a cultural care program, including main topics, educational objectives and contents, assignments and activities for students, teaching and evaluation methods. The curricular objectives and educational contents were implemented in five sessions to produce measurable results. The quantitative step showed that nursing students’ cultural competence in the intervention group (184.37 ± 22.43) improved significantly compared with the control group (153.19 ± 20.14) (t = 6.24, *p* = 0.001) after intervention.

**Conclusion:**

A cultural care training program can be designed by the model applied in this study in order to improve cultural competence of nursing students. This training program will be effective if students’ learning needs, appropriate assignments, and acceptable teaching methods are addressed. Therefore, nurse educators can design comprehensive training programs to improve nursing students’ cultural competence in different cultures and contexts. This training program is highly efficient because it is applicable in many disciplines of nursing education.

## Background

Culture and care are crucial for human survival. Understanding the cultural diversity and its components, such as distinct practices, beliefs and values, communication patterns, mental process, behaviors, traditions, and philosophy of clients can assist healthcare providers to respond in a culturally sensitive manner. Cultural diversity is used to implement healthcare services and programs. Now, healthcare systems are full of clients (families, individuals, communities, and populations) with varied cultures [1]. Changing population demographics and the cultural diversity necessitate examination and change of care processes; therefore, healthcare providers must provide culturally diverse nursing care. According to the American Nurses Association (ANA), cultural competence and professional growth is an important part of the nursing code of ethics. ANA introduced a new standard, culturally congruent care, to the Nursing: Scope and Standards of Practice, as the number of culturally and ethnically diverse clients increases [2, 3].

Leininger’s sunrise model can be used to culturally assess patients or clients, so nurses and other healthcare providers can identify worldview, cultural and social components of community, and also decision and action modes to provide culturally congruent care [1]. Culturally congruent care considers the client’s cultural values, beliefs, worldview, and lifeways, while making decisions about their health and well-being or preventing illness, disabilities, or death [4]. Culturally congruent care is evidence-based nursing that respects the healthcare consumer’s and other stakeholders’ cultural values, beliefs, worldview, and practices. As culturally congruent care encompasses all consumers, healthcare delivery systems, and nursing roles; therefore, practice evaluation ranges from fairly simple to complex. Culturally congruent care improves skills, behaviors, and practice of healthcare providers and patient satisfaction [5].

Cultural competence can provide culturally congruent care [5] and includes a set of congruent behaviors, attitudes, and policies that enable a system, agency, or professionals to work effectively in cross-cultural situations [6]. Campinha-Bacote’s model defines cultural competence consists of five components: cultural awareness, cultural knowledge, cultural skills, cultural encounter, and cultural desire [7]. Most nurses will achieve culturally congruent care and competencies through the lifelong professional development. This process begins with academic education and continues throughout the nurse’s professional life [5]. Therefore, nursing students must develop cultural competence during their studentship. However, cultural competence is not a major attitude in the nursing education. Nursing students may feel unprepared to deliver culturally competent care to culturally diverse clients [7].

Several descriptive studies conducted in different countries [8–11] and in Iran [12] found that nurses and nursing students had low to moderate levels of cultural competence. All studies emphasized the training programs to promote nursing students’ cultural competence. Interventional studies also revealed the effectiveness of training programs in promoting nursing students and graduates’ cultural competence [11, 13–15]. Several studies showed that cultural care education increased cultural safety among the healthcare team, nurses, and nursing students to prevent cultural bias, unhealthy, discriminatory, or degrading attitudes toward patients, and to build trust in patients and their families [16, 17]. A systematic review also found that nursing students considered lack of cultural competence and self-efficacy, and language as barriers to assessing patients from other cultures. Cultural encounters, knowledge, and experiences were the most important facilitators of cultural competence. Cultural diversity education, experience with taking care of patients from different cultures, and special populations were key factors influencing cultural competence. Researchers emphasized that nursing students’ cultural competence should be improved through cultural care education [7]. Undergraduate nursing students must develop cultural competence through undergraduate nursing curricula during their studentship. Effective strategies should integrate cultural competence into the undergraduate programs. However, there is limited research on the most effective method to promote cultural competence in undergraduate nursing students. This finding supports the literature that a culturally competent curriculum or experience will promote cultural competence in undergraduate nursing students [6]. Other researchers highlighted in a mixed methods study that the experiences and perspectives of students, nurse educators, and nurses should be studied using qualitative data in order to improve students’ cultural competence. The training program should then be tailored to the needs of the students and evaluated quantitatively [18]. Another study emphasized that nursing programs had to address educational need of students. Students’ involvement in training programs helps them realize their crucial roles in designing and developing curricular. Moreover, nurses and nurse educators’ varied approaches and perspectives strengthen the training programs. Therefore, educators can help future nurses provide care for multicultural populations better [18].

It is not clear how to teach cultural care to nursing students. We aimed to address this gap by designing, developing, implementing, and evaluating appropriate teaching and learning approaches in cultural care training program contextualized within a systematic model. The Talbot and Verrinder model in health education can be a guide to design, implement, and evaluate cultural care training program for promoting cultural competence among nursing students. Therefore, subjects for cultural care education must be identified to prepare nursing students for cultural competence. Additionally, the effects of this program on nursing students’ cultural competence can contribute to future cultural nursing education activities, strategies, tactics and educational contents. However, the literature does not adequately address training programs for improving nursing students’ cultural competence. Further rigorous mixed methods studies are needed to design and evaluate training programs in different contexts and cultures. Therefore, this study aimed to design, implement, and evaluate a cultural care-training program to improve cultural competence of undergraduate nursing students.

## Methods

### Study design and settings

This mixed study conducted with a sequential exploratory (qualitative-quantitative) method aimed to design, implement, and evaluate a cultural care-training program to improve cultural competence of undergraduate nursing students. The study setting was Razi School of Nursing and Midwifery affiliated to Kerman University of Medical Sciences in Southeastern Iran. The Talbot and Verrinder model with the following six steps was used to design this training program:

1. Identification and determination of a specific topic for planning (needs assessment)
2. Initial design of the program
3. Finalization of the program (using the views of experts in this area)
4. Implementation of the program
5. Implementation and monitoring of the program
6. Evaluation of the program [19].

**Fig. 1:**
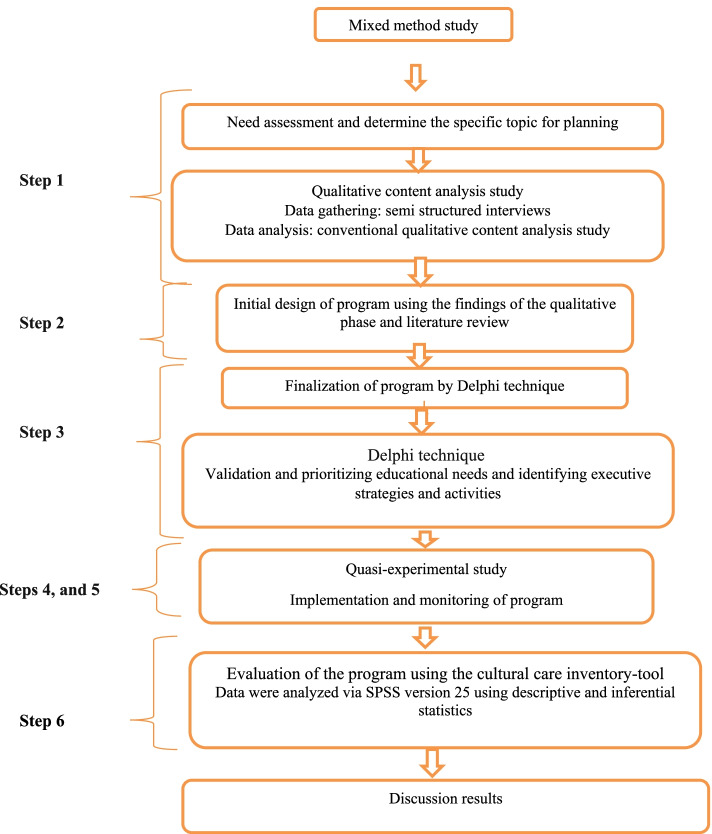
Flow diagram of the study

All steps were completed in the spring and summer 2021. The following features were taken into account to design and develop the cultural care training program. The study design is also summarized in Fig. 1.

#### Step 1: needs assessment for improving cultural competency (qualitative study)

According to the first step of the Talbot program, educational needs of students and a specific topic for planning were extracted and formulated using a conventional qualitative content analysis study. The purposive sampling was used to select 14 nursing students, 2 nursing faculty members from Razi School of Nursing and Midwifery, and 2 nurses working in a hospital. Inclusion criteria were nursing students, who had passed two-thirds of their clinical credits and the ability to communicate and express rich experiences, nursing faculty members, who actively were teaching theoretical and clinical courses (baccalaureate, master, PhD) and had Bachelor’s and higher degrees in nursing, nurses with at least one-year experience of mentorship for nursing students at clinical settings and with bachelor’s or higher degrees. The exclusion criterion was unwillingness to continue the study for any reason.

The corresponding author, a PhD student who had a background in nursing education, selected a number of eligible participants. She asked the nursing faculty members to introduce nursing students and nurses, who would be representative of their peers and to speak openly. The sampling process was based on maximum variation to obtain rich and diverse perspectives and experiences of the participants. Data were collected through open and semi-structured interviews as well as field notes. The corresponding author observed health protocols related to the Covid-19 pandemic and conducted face-to-face interviews or used the Skyroom videoconferencing platform. She invited and explained the study objectives to participants, and scheduled an interview. Informed consent was obtained from the participants for recording the interviews, and the time, length, and location of the interview were chosen based on the participant’s preference. The entire interview was recorded and converted into audio files. Simultaneously, she asked specific questions to ensure that the objectives were met. Here are some broad guideline questions to consider:

- Would you please share your experiences of the cultural components and cultural diversity of clients?
- Can you share your experience of caring for culturally diverse patients / clients?
- What training do you need to improve cultural competencies?
- What educational strategies do you suggest to improve students’ cultural competencies?

Graneheim and Lundman’s content analysis method was used to analyze the interviews in the qualitative phase. The four criteria of credibility, dependability, confirmability, and transferability were used to assure data trustworthiness [20].

#### Step 2: initial design of the cultural care-training program

Two actions were made to initialize the program (the second step in the Talbot program). First, an initial draft of the cultural care-training program was prepared using the findings of the qualitative phase. Broad goals, specific objectives, suggested training approaches, and activities required for the improvement of cultural competency of students were developed. Second, a brief literature review was conducted, so researchers could extract and understand cultural care curricula (content, training approaches, structure, materials, timing, and evaluation methods) [7, 21–30]. Moreover, the literature review showed how to integrate cultural care into nursing educational programs. Finally, the researchers prepared the initial draft of the program by prioritizing the needs and identifying executive strategies and activities.

#### Step 3: finalization of the cultural care training program (Delphi technique)

Finalization and validation of the program was equal to the third step of the Talbot program. This step was conducted using classic Delphi technique in two rounds and according to the priorities and objectives of the program. Using a purposive sampling, the first researcher selected five faculty members from the Razi School of Nursing and Midwifery and five faculty members from Nursing and Midwifery schools affiliated with medical universities of Kerman province (Rafsanjan, Jiroft, Bam, Sirjan, and Zarand). Inclusion criteria included faculty members, who had Master’s degree or PhD in nursing with scientific knowledge in the area of study and at least 2 years of work experience in teaching nursing students (baccalaureate, master, PhD) in practice and theory. An exclusion criterion was their unwillingness to continue the research.

The initial version of the cultural care training program that was prepared using the findings of the previous steps was submitted to experts via email in the first round. The expert panel’s recommendations were included into the subjects and objectives and the pattern of learning and teaching experiences was stabilized. They were asked to prioritize educational needs, suggestions, and strategies, rate each of the educational needs and approaches (ranging from one to three), their cost-effectiveness, ease of implementation and effectiveness. After gathering their opinions, the researchers organized executive strategies and activities, program content and timing. The panel of experts was invited to comment on the program’s strengths and weaknesses in the second round of Delphi. Finally, the curriculum of the cultural care training was revised and the final version was accepted (Table 3).

#### Steps 4 and 5: implementation and monitoring of the cultural care training program (quasi-experimental)

A quasi-experimental pilot study was done to evaluate the effectiveness of curriculum of the cultural care training**.** Using census, all senior undergraduate nursing students of Razi School of Nursing and Midwifery (*N* = 73) were enrolled in this step of study. According to Iranian academic law, a bachelor’s degree includes 4 years of study, including theoretical and clinical credits. In the final year, nursing internship program prepares senior students for professional nursing practice, who then work under supervision of nurse instructors in clinical settings. The school of nursing appoints a faculty of member from each educational department in accordance with the students’ credits to supervise the clinical education and resolve educational challenges of the students. It is noteworthy that the faculty members selected to design the program did not supervise and observe final-year students. Inclusion criteria were senior students willing to participate in the training program, who passed all clinical credits before the internship and passed the Objective Structured Clinical Examination (OSCE) regarding the nursing skills. Students, who were absent from more than one session, were on leave, or transferred to other universities and did not complete the questionnaires, were excluded.

To implement the program, the second researcher prepared a list of eligible senior students’ names and their cell phone numbers, and then randomly assigned them to intervention (*n* = 37) or control (*n* = 36) groups using randomization software. Due to the outbreak of COVID-19 disease, the researcher created two separate WhatsApp channels for the control and intervention groups, and electronically distributed the consent form and the pre-intervention questionnaire to the two WhatsApp groups. Additionally, necessary instructions for completing the questionnaires were supplied in the WhatsApp channels, and then the questionnaires were sent to the researcher using the Google Form link. The researcher cooperated with the intervention group to determine the time of training program.

The researchers trained cultural care in five two-hour sessions for 6 weeks. Training sessions were conducted online using the Skyroom platform because students were unable to attend college due to the COVID-19 pandemic. The researchers demonstrated how to manage the training program, how to create a schedule for the training program, and how to log into Skyroom. Additionally, students in the intervention group could access to audio files, PowerPoint presentations, video tutorials, and textual help offline via WhatsApp and the Navid Virtual Education System (https://kmunavid.vums.ac.ir/account/loginsama). As a result, participants could study materials and prepare for the next session. According to Table 3, students’ assignments and activities included investigation of a culture, cultural assessment of a client, writing a case report, and doing scenario projects. The second researcher used WhatsApp and SMS reminders to encourage students to access materials in Navid, as well as to complete assigned work and attend the next session. Students furthered course materials through self-study, completed hands-on exercise, and submitted assignments via E-mail, WhatsApp, or the Navid. The researchers sent feedback to the students via the Navid system and WhatsApp. Moreover, researchers closely monitored the study conditions to ensure that the program was implemented according to the curriculum, students completed assigned homework, and that students attended training sessions.

#### Step 6: evaluation of the cultural care-training program using the cultural care inventory

Two tools were used to collect data in the evaluation step. The demographic and academic information questionnaire included the students’ age, gender, marital status, ethnic background, history of attending in cultural care training course, etc. (Table 2).

Nursing students’ cultural competence was evaluated using cultural care inventory designed by Moulder (2009) as an adaptation from models of Campinha-Bacote, Leininger, and Purnell. There are 51 items in four subscales: cultural awareness (11 items), attitudes toward cultural care (9 items), preparation in cultural care (19 items), and cultural skills (12 items). Items are rated on a 5-point Likert scale, with five indicating strongly agree and one indicating strongly disagree. The scores vary from 51 to 255. Total score between 51 and 109 was considered as poor cultural competence, the total score between 110 and 167 was considered as moderate cultural competence and the total score between 168 and 225 was considered as a high level of cultural competence. The validity of the original inventory was confirmed by an expert panel, and its reliability was corroborated using the internal consistency method; Cronbach’s alpha was calculated to be 0.77, 0.76, 0.77, and 0.91 for the cultural awareness, attitude towards cultural care, skills in cultural care, and preparation in cultural care subscales, respectively [31].

The validity and reliability of the Persian version of cultural care inventory were confirmed by Bastami et al. in Iran. A panel of experts confirmed content validity of the inventory, and its reliability was established by internal consistency. The Cronbach’s coefficient for the whole scale was reported to be 0.86 [32]. The validity and reliability of this inventory have been confirmed in several studies in Iran [33–36].

One month after implementing training program, the questionnaire’s link was sent to WhatsApp groups, and the participants completed the questionnaires. Nursing students were expected to apply the competences they learned in clinical settings and real situations after 1 month.

### Statistical analysis

Quantitative data were analyzed via SPSS25, descriptive statistics (frequency, percentage, mean and standard deviation), and inferential statistics (independent samples t- test, paired t- test, chi-square, analysis of covariance, and multivariate linear regression). Level of significance was considered *P* < 0.05.

## Results

### Demographic and academic information

The first step was done through 14 interviews with senior nursing students, two interviews with nursing faculty members, and two interviews with nurses. The mean age of the participants was 28.9 years, and the mean work experience of nursing faculty members and nurses was 17.2 years. Eleven participants were female and the rest were male (Table 1).

**Table 1:** Demographic and academic information of interviewees in the qualitative study (step1) and expert panel of Delphi technique (step3).

Variables	Groups	Interviewees (***n*** = 18)		Expert panel (***n*** = 10)	
		*n*	%	*n*	%
Gender	Female	11	61.1	8	80
	Male	7	38.9	2	20
Marital status	Single	13	72.2	1	10
	Married	5	27.8	9	90
Degree	undergraduate	14	77.8	–	–
	Bachelors (BA)	2	11.1	–	–
	Masters (MS)	0	0	1	10
	Ph.D.	2	11.1	9	90
Position	Student	14	77.8	–	–
	Clinical nurse	2	11.1	–	–
	Faculty member	2	11.1	10	100
		**M ± SD**		**M ± SD**	
Age (years)		28.9 ± 10.1		45.7 ± 8.2	
Work experience (years)		17.2 ± 6.1		19.5 ± 7.1	

In the step 3, 10 nursing faculty members completed two rounds of Delphi technique (response rate = 100%). The mean age and work experience of participants were 45.7 years and 19.5 years, respectively. Eight participants were female, of whom nine had PhD (Table 1).

Table 2 shows demographic and academic information of the nursing students in the evaluation step of the program. Seventy-three senior undergraduate nursing students completed the study (100% response rate). The mean ages of students in the intervention and control groups were 20.8 and 21.4 years, respectively. Most of the students in the intervention and control groups were female (64.9, 58.3%), single (97.3, 83.3%), Kerman natives (81.1, 66.7%), and had no history of attending in cultural care training course (97.3, 97.2%). In addition, the majority of students in the intervention and control groups believed that the present nursing curriculum did not provide cultural care training (91.9, 88.9%). The majority of students in the intervention and control groups moderately cared for culturally diverse people in the clinical setting (59.5, 58.4%).

**Table 2:** Comparison of demographic and academic information between the intervention and control groups in the evaluation step

Variables	Groups	Intervention		Control		χ^**2**^	***P***- value
		***n***	%	***n***	%		
Gender	Male	13	35.1	15	41.7	0.33	0.57
	Female	24	64.9	21	58.3		
Marital status	Single	36	97.3	30	83.3	4.1	**0.04**
	Married	1	2.7	6	16.7		
Ethnicity	Kermani	30	81.1	24	66.7	6.74	0.34
	Fars	5	13.5	6	16.7		
	Baloch	1	2.7	0	0		
	Turkish	0	0	3	8.3		
	Lor	0	0	1	2.8		
	Others	1	2.7	2	5.5		
History of attending in cultural care training course	No	36	97.3	35	97.2	0	0.98
	yes	1	2.7	1	2.8		
Status of training courses of cultural care in curriculum	None	34	91.9	32	88.9	4.04	0.13
	1-2 course	3	8.1	1	2.8		
	Integration throughout the course	0	0	3	8.3		
The level of care for people with cultural diversity during clinical education	Low	3	8.1	7	19.4	2.41	0.30
	Moderate	22	59.5	21	58.4		
	High	12	32.4	8	22.2		
		**M ± SD**		**M ± SD**		**Independent** **t- test**	***P*** **-value**
Age		20.8 ± 1.27		21.4 ± 2.81		−1.51	0.13

No significant difference in demographic and academic information was found between the intervention and control groups except for marital status.

### Main results

Six steps of the Talbot and Verrinder’s model were applied in this study for designing, implementing, monitoring, and evaluating the cultural care-training program (Fig. 1).

#### Steps 1.2.3

With needs assessment process, the researchers were familiar with the academic and clinical contexts, educational contents, activities, and methods in cultural care education, as well as students’ learning needs. The needs assessment also included experiences and suggestions from nurse educators, nurses, and students, as well as case studies and scenarios they considered useful in designing cultural competence of students. These examples were beneficial to reinforce the training by relating them to lived experiences of participants while providing cultural care. Additionally, literature review and Delphi technique were used to modify the identified needs as well as to correctly integrate them in cultural care curricula. Moreover, an expert panel assessed the curricula to ensure that the training topics and objectives, contents, methods, and assignments were relevant and well-planned in order to achieve cultural competence aims. Assignments were designed to help students achieve the program objectives. The final curriculum had five broad topics and a single goal. Table 3 shows the curriculum of the cultural care training.

**Table 3:** Curriculum of the cultural care training

Goal: To improve cultural competence of senior undergraduate nursing students			
**Target group:** undergraduate nursing students in intervention group (n = 37)		**Educational methods:** Tutorials and lectures PowerPoints Case reports and Scenario Educational video and video clip, audio-visual material Interactive forum and discussion Role playing Web-based learning Oral reports and storytelling Expression of work-based experiences and self-reflection Presentation of articles and their important points Book reading Questions and answers	**Evaluation method:** Questionnaire and comparison of pre-test and post-test scores
**Expected duration:** 5 two-hour sessions in 6 weeks			
**Sessions**	**Topics**	**Objectives and educational contents**	**Assignment for students**
1	- An introduction to multicultural education - Familiarity with culture and components of cultural diversity	- The aims of multicultural education in higher education and nursing - Definitions of culture, ethnicity, nationality, and race - Culture iceberg model and components of culture -Understanding of cultural history, cultural diversity, factors affecting cultural diversity such as biological variations, age, religion, nutrition, social and economic context, language, roles related to sexual identity, etc. - Culture, ethnicity and cultural subgroups in Iran and cultural diversity in southeastern Iran - Group history, health status, and epidemiology of minority groups - Bio-physiological determinants of health, and illness of minority groups -The underlying factors and the impact of race/ethnicity, culture, and class on clinical decision-making - Identification of healing traditions and beliefs of clients at the workplace and education	- Book reading about culture of Kerman and southeastern Iran - Investigation of a culture and cultural diversity in clients of a clinical setting and a health center daily -Writing of a work-based experience with assessment of patient’s background when providing care - Writing of brief project to assess culture of an individual, family, and/or community to appreciate the specific and universal aspects of culture - Provision of the autobiography assignment to explain that everything we do emanates from who we are
2	-Cultural care and nursing process -Cultural competence in the organization	- Definition of cultural care or culturally Congruent Care, and cultural competence (individual, system, or organizational) -Prerequisites for cultural competence - The importance of creating cultural competence among nursing students -Integration of culturally congruent care into the stages of nursing process: Assessment: LEARN model, BELIEF model, 4 c model and documentation of clients’ health beliefs/model of their illness and health Nursing diagnoses Implementation of interventions Evaluation of outcomes - Cross-cultural communications and a model for effective cross-cultural communications, data gathering and documentation - Standards for culturally congruent care - Organizational cultural competence - Analysis of cultural competency issues in health systems and organizations -Strategies and barriers to organizational competency (clinical, organizational and structural) - Demonstration of respect for a patient’s cultural and health beliefs -The importance of curiosity, empathy, and respect in patient care - National documents/standards - Healthy People 2020 etc. for culturally congruent care - The importance of diversity in health care as well as its challenges and opportunities	- Doing scenario project - Designing a plan of care based on inferences that are culturally meaningful and congruent with the patient’s culture - Assignment addressing integration of cultural care into nursing process with a case study: - Cultural assessment of a client with interview using LEARN model and cultural interview report -Nursing diagnoses, implementation of interventions, and evaluation for the case study - Reading educators’ suggested articles -Writing viewpoints on a video about culturally cross-cultural communication presentation and discussion
3	Models of cultural care and process of gaining cultural competence in nursing	- Comparing and applying models of effective cultural care: - Leininger’s Sunrise model (culture care preservation/maintenance, culture care accommodation/negotiation, culture care re-patterning / restructuring, culture brokering ( -Giger and Davidhizar’s evaluation model (communication, space, social organization, time, environmental control, and biological variations) -Purnell’s model of cultural competence (culture or heritage, communication, family and organization roles, labor issues, high-risk behaviors, nutrition, pregnancy, death, spirituality, health care measures, and health care professionals) - Campinha–Bacote’s model (cultural awareness, cultural knowledge, cultural skills, cultural exposure, and cultural desire) - Orlandi’s model (cognitive, affective, and psychomotor domains in cultural competence), cultural sensitivity and incorporation of culturally sensitive approaches to nursing care -Respect for a patient’s culture and health beliefs and use of negotiating and problem-solving skills in decision-making shared with a patient	- Doing scenario project - A case study addressing actions and interventions of cultural care based on Leininger’s Sunrise model - A case study addressing assessment of a client based on Purnell’s model - Reading educators’ suggested articles -Writing viewpoints on a video presentation
4	-Cultural competence outcomes -Barriers and challenges of cultural competence	- Positive consequences of cultural competence and cultural care delivery -Equality of health, social justice, health literacy, - Barriers to cultural care and recognition of potential biases affecting clinical encounters, clinical decision-making and quality of care: (stereotyping, prejudice, discrimination, racism, ethnicity, cultural destructiveness, cultural imposition, cultural conflict, cultural shock, cultural blindness, cultural incapacity) - Description of access method, historical, political, environmental, and institutional factors - Barriers to effective patient care, such as diagnostic inaccuracies, unintentional patient exploitation, racial and ethnic inequalities and disparities, group communication difficulties, diagnostic inaccuracies, and unintentional patient exploitation. -Data on health disparities in community, state, or in the nation and provision of information about local community leader(s) and community groups/resources serving this community	-Case study and writing information on how to acknowledge personal biases, patient stereotyping, assumptive bias and the confounding of ethnicity with socioeconomic status, and their effects on the client’s health - Data review: - Review of an article - National Healthcare Disparity Reports -Writing viewpoints on a video presentation
5	-Providing cultural care to immigrants -Summary, questions and answers	- Migration and cultural diversity in immigrants -Immigration status and conditions - Complementary medicine and common diseases among immigrants -Process of acculturation in immigrants -Patterns of cultural acculturation: cultural assimilation, separation, integration, marginalisation -Culturally congruent care for immigrants -Strategies for health education programs with cultural competence for immigrant clients - Summary and conclusions - Question and answer	- Assignment addressing culturally competent care on an immigrant client with case study-based learning, cultural reading literature - Writing a scenario for an immigrant client - Reading educators’ suggested articles -Writing viewpoints on a video presentation

#### Steps 4 and 5

The curricular objectives and educational contents were divided into five sessions to achieve measurable results. Moreover, educational methods or strategies, practical activities, and assignments for the nursing students were used according to the program. First, the researchers presented training program visually in a slide presentation and discussed how their cultural competence was related to their expectations and objectives. Then, the researchers facilitated the training by creating a friendly, trustful, and respectable atmosphere. They instilled in the students a sense of self-discipline and a desire to learn, and prepared them to participate actively in the training program because a successful training depended on the activities that students participated. Students were encouraged to share their expectations, opinions, and experiences in each session. Students were able to get answers to any questions concerning the training, materials, and assignments using online communication channels.

#### Step 6

Researchers evaluated the effectiveness of curriculum using cultural care inventory. Table 4 shows the level of cultural competence in both groups before and after the intervention. The mean pre-intervention scores of cultural competence in the intervention group (151.89 ± 26.88) and the control group (151.8 ± 25.12) were moderate and had no significant difference (t = 0.01, *p* = 0.99). However, the mean post-intervention score of cultural competence in the intervention group (184.37 ± 22.43) increased significantly compared with the control group (153.19 ± 20.14) (t = 6.24, *p* = 0.001), so that cultural competence in the intervention group promoted from the “moderate” to “high” level.

**Table 4:** Comparison of scores of cultural competences between the control and intervention groups before and after the training program

	Time	Pre-intervention	Post- intervention			
**Variables**	**Groups**	**M ± SD**	**M ± SD**	**Mean difference**	**Paired** ***t*** **-test**	***P*** **- value**
Cultural awareness	Intervention	32.7 ± 5.69	39.43 ± 4.3	6.7	−7.09	**0.001**
	Control	32.44 ± 6.92	33.5 ± 4.67	1.4	1.03	0.31
	Independent *t*- test	0.19	5.64			
	*P*-value	0.85	**0.001**			
Attitude towards cultural care	Intervention	33.48 ± 3.76	34.89 ± 2.89	1.40	−2.5	**0.02**
	Control	33.27 ± 3.96	34.27 ± 3.40	9.26	−1.52	0.14
	Independent *t*- test	0.23	0.83			
	*P*-value	0.818	0.41			
Cultural skills	Intervention	30.40 ± 7.99	39.43 ± 7.95	9.02	7.48	**0.001**
	Control	31.58 ± 8.59	30.94 ± 5.99	0.63	0.48	0.63
	Independent *t*- test	−0.61	5.13			
	P-value	0.55	**0.001**			
Preparation in cultural care	Intervention	55.27 ± 13.42	70.62 ± 12.31	15.35	−7.39	**0.001**
	Control	54.5 ± 11.62	54.47 ± 12.10	0.02	0.014	0.99
	Independent *t*- test	0.26	5.64			
	*P*-value	0.79	**0.001**			
Total of cultural Competence	Intervention	151.89 ± 26.88	184.37 ± 22.43	32.48	−8.50	**0.001**
	Control	151.8 ± 25.12	153.19 ± 20.14	1.38	−0.37	0.71
	Independent *t*- test	0.01	6.24			
	*P*-value	0.99	**0.001**			

Bold *p*-values are significant at level of ≤0.05

Covariance analysis test was used to confirm the results of Table 4, showing that by controlling the impact of pretest and marital status on the cultural care competence, the total posttest scores of cultural care competence were significantly different between the intervention and control groups (*p* = 0.001) **(**Table 5**).**

**Table 5:** Summary of covariance analysis for the two groups of intervention and control

		df	Mean square	F	***p***-value
Cultural Competence	Corrected model	3	9181.27	28.12	**0.001**
	Intercept	1	13,196.14	40.42	**0.001**
	Marital status	1	14.73	0.04	0.83
	Pre-intervention	1	16,870.43	21.71	**0.001**
	Group	1	16,451.77	50.39	**0.001**
	Error	69	326. 46		

Bold *p*-values are significant at level of ≤0.05

In addition, the multivariate linear regression (Forward stepwise method) was conducted to eliminate the impact of confounding variables (such as age, gender, marital status, ethnicity, history of attending in cultural care training course, status of training courses of cultural care in curriculum, and the level of care for people with cultural diversity during clinical education) on the cultural competence. The results showed that these variables were not significant predictors of cultural competence in nursing students in this study (Table 6).

**Table 6:** Multivariate regression model for confounding variables and cultural competence

	Unstandardized coefficients		Standardized coefficients		
	B	Standard error	B	t	***p***-value
Constant	194.12	30.76		6.32	**0.001**
Age	−0.82	1.32	−0.069	− 0.62	0.53
Gender	−4.51	5.14	−0.084	−0.87	0.38
Marital status	1.74	9.19	0.02	0.18	0.85
Ethnicity	3.90	1.79	0.19	0.17	0.33
History of attending in cultural care training course	3.70	1.71	0.06	0.58	0.56
Status of training courses of cultural care in curriculum,	3.95	1.29	0.01	0.01	0.47
The level of care for people with cultural diversity during clinical education	3.04	1.03	0.02	0.24	0.28
Time	3.10	1.42	0.1	0.42	0.18
Group	−33.38	4.87	−0.63	−6.84	**0.001**

Bold *p*-values are significant at level of ≤0.05

## Discussion

This study aimed to design, implement, and evaluate a cultural care training program for improving cultural competence of undergraduate nursing students. Designing this program based on the documentations of the qualitative study, review of the literature, and expert panel could improve the cultural competence of undergraduate nursing students.

In support of our results, researchers acknowledged that health educators frequently used psychosocial models when designing, implementing, and evaluating outcomes of curriculum and health education interventions. Models serve as a starting point for exploring clients’ perspectives, beliefs, values, and practices. After understanding clients’ viewpoints, practitioner and clients can collaborate more effectively to find workable solutions. Incorporating models helps students develop cultural competence [23]. Moreover, Tessier et al. (2021) suggested that needs assessment was critical for familiarity of instructors with the organizational context, appropriate educational contents, understanding of participants’ practical experiences, and the value of training. It can assist instructors in identifying participants’ main concerns and experiences to enhance the effectiveness of training programs. Such needs assessment also helps instructors establish a conducive learning environment by fostering relationships with participants [37]. Creswell and Guetterman (2019) also believe that people are the best source of describing their own situation, cultural context, needs, feelings, and experiences [38]. Researchers in another study emphasized that cultural competence education should be congruent concerning the needs, the levels of receptivity of the learners, as well as context, culture, policies and guidelines, and accreditation of educational institutions. Diverse patients, community representatives, consumers and advocates should participate in the design, implementation, and evaluation of cultural competence curricula whenever possible [39].

In addition, evidence suggests that cross-cultural learning can be improved by reconsidering educational philosophies and learning and teaching methods in theory and practice as well as exploring the benefit of cultural care. Therefore, students can communicate with individuals from other cultures [40, 41]. Using a literature review and consultation with academics, Allen et al. (2013) developed the teaching-learning methods and learning objectives for nursing students’ cultural care education. The teaching-learning method was modified with experiential learning and role-playing. Then, they evaluated students’ learning through quantitative study using pre and post-test. The study findings supported the effectiveness of this strategy in promoting students’ confidence in cultural nursing care [21]. Two meta-analyses revealed that the best way to educate cultural competence was through a variety of educational strategies, including lectures, in-depth, interactive exercises and discussions, case study analysis, genograms, presentation of articles, selected readings and web-based learning and data collection, videos, simulations, role-playing, seminar, in-service-based learning, poster presentations, interview with clients, and development of a measuring tool [26, 42]. Researchers systematically reviewed 11 studies to evaluate cultural care training program. The results showed that cultural competence interventions/programs were integrated into core theoretical courses, or offered as elective courses. Debates, discussions, case scenarios, practicums, simulation, international learning projects, experiential learning, storytelling, and lectures were among the various types of teaching methods. Ten studies reported a statistically significant increase in the level of cultural competencies. Educational content, teaching methods, and training courses were not uniformly described in the literature. More comprehensive, valid, and reliable measurement tools are needed to evaluate the education provided for nursing students [7].

In the quantitative part of this study, the training program was piloted and evaluated in a group of nursing students. After the implementation of the cultural care training program, cultural competence in the intervention group increased significantly compared with the control group. Cultural competence in the intervention group was promoted from “moderate” level to “high” level. Consistent with our results, several interventional studies evaluated and validated the effectiveness of cultural care training programs with cultural competence questionnaires [13, 23, 43–48]. Bauer and Bai (2018) used a model to develop a curriculum to enhance cultural competence among students of Nutrition and Food Science. They selected subjects, assignments, and activities based on a review of publications and websites from governmental, educational, and professional organizations. Evaluation of pre- and post-test data showed a significant improvement in students’ cultural competence [23]. Researchers in another study reported that cultural competence of senior oral health students increased significantly after receiving cultural competence training. They emphasized that cultural competence is a multifaceted structure, and an online training program is not enough to meet the needs of culturally diverse patients. Clinical work experiences and the community where students study help them to strengthen their level of cultural competence. In addition, patient feedback and health outcomes are important components of cultural care learning [49]. Lin et al. (2015) in one study conducted cultural care training and found that the cultural competence of nursing students improved; however, the overall effectiveness of the training decreased over time and in follow-up. The researchers stressed that cultural care training and assessment of its effectiveness in students and nurses’ learning should be done continuously [22].

Singleton (2017) demonstrated that after completing the cultural care program, PhD nursing students in New York raised their cultural competence and self-efficacy. According to the researcher, American Nursing PhD curricula were acceptable and sufficient in accomplishing national and professional goals to improve the practice of culturally competent nurses since these programs promoted learning based on societal requirements and demands to provide cultural care [50]. Our research suggests that nursing researchers examine the curriculum of undergraduate and postgraduate nursing students in Iran to achieve national and professional goals, equip students with appropriate cultural competence, and develop a program based on the needs of these target groups and the needs of a multicultural society. Researchers argue that most nurse educators teach cultural care to students and learners through a discontinuous training program that is limited to theoretical topics. Although these training programs can help increase cultural competence, they are insufficient for long-term motivation, attitudes, and behavior change after graduation. Nurse educators and students should pay special attention to cultural competence as a lifetime process, and participation in training programs is required even after years of working in the clinical settings [44]. Above-mentioned studies may be consistent with the present study because of new and diverse educational content and duration of training.

However, two studies showed that cultural care training in the form of cross-cultural communication workshops [24], simulation-based educational method, and in-service-based learning project were not effective in improving participants’ cultural care competence [51]. They suggested the need to develop and organize cultural training programs more coherently to increase the number of programs with fewer participants.

The findings of study have implications for curriculum developers and nurse educators. Cultural competence must be an important outcome for graduate nursing students. In order to prepare nursing students to enter the clinical settings, cultural competence should be considered in undergraduate nursing education. For this purpose, it is necessary to change the perspective, increase the cultural competencies of nurse educators, and create organizational cultural competence. Moreover, cultural competence must be used as a key part of the professionalization process in various fields of nursing, including education, clinic, community, research and management. Therefore, this requires proper context, adoption of long-term strategies, spending of more time and energy and understanding of the multicultural approaches by policy makers, curriculum designers, nurse educators, and educational managers in nursing schools. This study helps nurse educators to focus specifically on educational specific needs of undergraduate nursing students and teaching and learning approaches to improve cultural competence students using a simple and effective method. The use of a specific model makes the work of an instructional designer more systematic. To address cultural care in the design of health education programs and community health nursing education, Talbot and Verrinder (2005) model, which is more relevant to cultural competence, was used in this study to design the training program. Models act as a guide to curricular development. No curriculum model alone is sufficient for the entire curriculum of an educational center that educates a diverse population with multifaceted goals. Curricular designers and developers need to be aware of existing models to make intelligent decisions about their designs. The instructional design models were employed by taking into consideration the following principles: instructional designs are systematic and reflective processes through which instructional principles are used in the teaching and learning programs by differentiating materials, resources, activities, and evaluation. In instructional design processes, the designers are required to know and inquire about the theories of learning, systematic analysis of learners, and management methods to enhance their ability to employ information technology and evaluate the teaching and learning processes appropriately. An appropriate instructional design model should guide the professional designers to form the instructions according to the learners’ viewpoints rather than from the content perspective. As a result, considering the learners, aims (or learning attainments/outcomes), methods, and evaluations is of great importance in instructional design processes [52]. A common message behind all the instructional design models maintains that various types of learning require various learning conditions. Given that the instructional design models are adopted based on various learning needs, designers try to match the requirements pertinent to each learning situation with the responding models during the design process. Although there are many instructional design models, the majority of these models consist of these essential phases ADDIE: analysis, design, development, implementation, and evaluation [53]. Given the continuous social, environmental and cultural changes in the nursing schools, offering a credible model is not possible for designing training programs. In this regard, nurse educators with higher levels of awareness about different instructional design models are better equipped to choose a model that is appropriate to their classes and courses. In-service training (professional development opportunities) is needed because each of the nurse educators must be well-trained in new century skills [52]. Novice instructional designers should be trained in specific communication and even project management skills. Education and training provide them with knowledge and skills on how to operate in specific situational contexts effectively [54].

### Limitations and strengths

The present study had several limitations. Study samples in the quantitative phase of the study were senior undergraduate nursing students from a nursing school in southeastern Iran, so the results are not generalizable. Moreover, we evaluated the effectiveness of the training program using a self-report inventory. We did not also ask specific questions about their learning experiences and viewpoints following a cultural care training program in a qualitative survey. Future studies can evaluate the effectiveness and usefulness of the cultural competence curriculum using a variety of qualitative and quantitative methods such as written test, self-assessment, and the 360-degree evaluation by students, educators, and managers, as well as practical tests. Finally, we collected data of evaluation step in two stages with an interval of 1 month due to the project’s financial and time constraints. A longer time may be required to get stronger results. The 3–6 month follow-ups and experimental studies should be conducted with a larger group and control of the confounding variables to determine whether cultural care training program has long-term effects on the cultural competence of nursing students.

Given the strengths of the present study, our experiences in designing, implementing, and evaluating this virtual cultural care training program based on a systematic model can be beneficial for developing future curricula aimed at fostering cultural competence of nursing students and other healthcare providers. This program was developed in response to the educational needs of Iranian undergraduate nursing students. Therefore, considering the cultural structure of Iran, the program can be used in similar cultural structures.

## Conclusion

This study described the usability of the Talbot and Verrinder’s model to design, implement, and evaluate a cultural care training program aimed at improving nursing students’ cultural competence. This systematic model helped to integrate experiences and viewpoints of students, nurse educators, nurses, and experts into research evidence for assessing the educational needs of students, organizing detailed educational content, assignments and activities for students, selecting teaching and evaluation methods in the curriculum. Teaching methods were effective, and evaluation of data revealed the effectiveness of this form of program in improving students’ cultural competence. This program was an example of the design and development a cultural care curriculum and contributed to the literature on cultural competence in nursing education. Moreover, these findings can assist nurse educators to design effective cultural care training and education using systematic models to better prepare future nurses to provide culturally congruent care to multicultural clients. Further studies are also suggested to assess cultural competence in healthcare providers and evaluate effectiveness of different approaches and models to enhance their competence in different cultures and contexts.

## Data Availability

The data are available upon request to the corresponding author after signing appropriate documents in line with ethical applications and decision of the Ethics Committee.

## Abbreviations

ANA: American Nurses Association
OSCE: Objective Structured Clinical Examination

## References

[1] Salinda MT, Hipona JB, Ilarde M, Tuazon A (2021). A concept analysis on culturally congruent care. Int J Nurs Pract.

[2] Association Nursing American. Nursing: scope and standards of practice: MD: Nursesbooks.org; 2015.

[3] Moss E, Seifert PC, O’Sullivan A. Registered nurses as interprofessional collaborative partners: creating value-based outcomes. Online J Issues Nurs. 2016;21. 10.3912/OJIN.Vol21No03Man04. 10.3912/OJIN.Vol21No03Man04 27857055

[4] McFarland MR, Wehbe-Alamah HB. Leininger’s theory of culture care diversity and universality: an overview with a historical retrospective and a view toward the future. J Transcult Nurs. 2019;30(6):540–57. 10.1177/1043659619867134 31409201

[5] Marion L, Douglas M, Lavin MA, Barr N, Gazaway S, Thomas E, et al. Implementing the new ANA standard 8: culturally congruent practice. Online J Issues Nurs. 2016;22(1). 10.3912/OJIN.Vol22No01PPT20. 10.3912/OJIN.Vol22No01PPT20 28493662

[6] Byrne D (2020). Evaluating cultural competence in undergraduate nursing students using standardized patients. Teach Learn Nurs.

[7] Tosun B, Yava A, Dirgar E, Şahin EB, Yılmaz EB, Papp K, et al. Addressing the effects of transcultural nursing education on nursing students’ cultural competence: a systematic review. Nurse Educ Pract. 2021;103171. 10.1016/j.nepr.2021.103171. 10.1016/j.nepr.2021.103171 34388616

[8] Kardong-Edgren S, Cason CL, Brennan MW, Reifsnider E, Hummel F, Mancini M, et al. Cultural competency of graduating BSN nursing students. Nurs Educ Perspect. 2010;31(5):278–85. 21086864

[9] Yang SY, Lim HN, Lee JH (2013). The study on relationship between cultural competency and empathy of nursing students. J Korean Acad Soc Nurs Educ.

[10] Seo YS, Kwon Y-C (2014). Factors influencing to the cultural competence in nursing students. J Converg Inf Technol.

[11] Gower S, Duggan R, Dantas JA, Boldy D (2019). One year on: cultural competence of Australian nursing students following international service-learning. J Nurs Educ.

[12] KHanbabayi-Gol M JF, Zamanzadeh V Cultural competence among senior nursing students of medical university in north-west Iran The J Urmia Nurs Midwifery Fac. 2017;15(8):612–19.

[13] Creech C, Filter M, Wehbe-Alamah H, McFarland MR, Andrews M, Pryor G (2017). An intervention to improve cultural competence in graduate nursing education. Nurs Educ Perspect.

[14] Choi KS, Lee SY, Park Y, Jun M, Choi J (2016). Development and an evaluation of educational program for nurse professionals: cultural competency in Cancer prevention. Asian Oncol Nurs.

[15] Bauer K, Bai Y (2015). Innovative educational activities using a model to improve cultural competency among graduate students. Procedia Soc Behav Sci.

[16] Pimentel J, Zuluaga G, Isaza A, Molina A, Cockcroft A, Andersson N. Curriculum Co-design for Cultural Safety Training of Medical Students in Colombia: Protocol for a Qualitative Study. In: World Conference on Qualitative Research, vol. 2018: Springer; 2018. p. 102–9.

[17] Bozorgzad P, Peyrovi H, Vedadhir A, Negarandeh R, Esmaeili M (2017). A critical lens on patient decision-making: a cultural safety perspective. Nursing and Midwifery Studies.

[18] Smit EM, Tremethick MJ (2013). Development of an international interdisciplinary course: a strategy to promote cultural competence and collaboration. Nurse Educ Pract.

[19] Talbot L (2018). Promoting health : the primary health care approach 6E / Lyn Talbot, Glenda Verrinder.

[20] Graneheim UH, Lundman B (2004). Qualitative content analysis in nursing research: concepts, procedures and measures to achieve trustworthiness. Nurse Educ Today.

[21] Allen J, Brown L, Duff C, Nesbitt P, Hepner A (2013). Development and evaluation of a teaching and learning approach in cross-cultural care and antidiscrimination in university nursing students. Nurse Educ Today.

[22] Lin C-J (2015). Chang P-r, Wang L-H, Huang M-C: cultural competence course for nursing students in Taiwan: a longitudinal study. Nurse Educ Today.

[23] Bauer K, Bai Y (2018). Using a model to design activity-based educational experiences to improve cultural competency among graduate students. Pharmacy.

[24] Majda A, Zalewska-Puchała J, Bodys-Cupak I, Kurowska A, Barzykowski K (2021). Evaluating the effectiveness of cultural education training: cultural competence and cultural intelligence development among nursing students. Int J Environ Res.

[25] Jang SM, Kim J (2018). Current status of transcultural nursing education in nursing baccalaureate programs. J Korean Acad Nurs.

[26] Gallagher RW, Polanin JR (2015). A meta-analysis of educational interventions designed to enhance cultural competence in professional nurses and nursing students. Nurse Educ Today.

[27] Hamidizadeh K, Fathivajargah K, Arefi M, Mehran G. Multicaltural education: systematic analysis of teaching perceptions. Res Teach. 2017;5(2):1–16.

[28] Sit A, Mak AS, Neill JT (2017). Does cross-cultural training in tertiary education enhance cross-cultural adjustment? A systematic review. Int J Intercult Relat.

[29] Tulman L, Watts RJ (2008). Development and testing of the blueprint for integration of cultural competence in the curriculum questionnaire. J Prof Nurs.

[30] Clark L, Calvillo E, Dela Cruz F, Fongwa M, Kools S, Lowe J, Mastel-Smith B (2011). Cultural competencies for graduate nursing education. J Prof Nurs.

[31] Moulder M. Senior nursing student level of preparation, attitudes, awareness, and competence in ethnocare: Dowling College; 2009.

[32] Bastami MR, Kianian T, Borji M, Amirkhani M, Saber S (2016). Assessment of cultural competence among nurses. J Med Ethics.

[33] Salehi Asl MA, Zargar SA, Fesharaki M: Evaluation of the relationship between cultural competence and job burnout of nurses. J Adv Pharm Res. 2019;9(S2):17–162.

[34] Firoozi F, Mozaffari N, Iranpour S, Molaei B, Shamshiri M (2020). The status of cultural care among nurses working in different wards of teaching hospitals in Ardabil, Iran: a cross-sectional survey study. Int J Care Coord.

[35] Khodaveisi M, Davodi L, Mohammadi Y, Mohammadi N (2018). Correlation between cultural competence and accountability in nurses working in hospitals in Hamadan. J Biochem Technol Special Issue.

[36] Mafakheri S, Anboohi SZ, Borhani F, Kazemi E (2020). Studying the correlation of nurses cultural competency and patient satisfaction in intensive care unit of hospitals affiliated to Kurdistan University of Medical Sciences in. Arch Pharm Pract.

[37] Tessier A, Croteau C, Voyer B. Exploring the usability of the andragogical process model for learning for designing, delivering and evaluating a workplace communication partner training. J Workplace Learn. 2021:577–90.

[38] Creswell JW, Guetterman TC. Educational research: planning, conducting, and evaluating quantitative and qualitative research: Pearson; 2019.

[39] P Gilbert MJ: Principles and recommended standards for cultural competence education of health care professionals. The Endowment 2003 https://www.mghihp.edu/sites/default/files/about-us/diversity/principles_standards_cultural_competence.pdf.

[40] Markey K, Okantey C (2019). Nurturing cultural competence in nurse education through a values-based learning approach. Nurse Educ Pract.

[41] Hovland OJ, Johannessen B. Nursing students develop cultural competence during student exchanges in Tanzania. Norwegian J Clin Nurs/Sykepleien Forskning. 2019;13:1–17.

[42] Zhang X, Zhou M (2019). Interventions to promote learners’ intercultural competence: a meta-analysis. Int J Intercult Relat.

[43] Fair F, Soltani H, Raben L, van Streun Y, Sioti E, Papadakaki M, Burke C, Watson H, Jokinen M, Shaw E (2021). Midwives’ experiences of cultural competency training and providing perinatal care for migrant women a mixed methods study: operational refugee and migrant maternal approach (ORAMMA) project. BMC Pregnancy Childbirth.

[44] Arruzza E, Chau M (2021). The effectiveness of cultural competence education in enhancing knowledge acquisition, performance, attitudes, and student satisfaction among undergraduate health science students: a scoping review. J Educ Eval Health Prof.

[45] Lin M-H, Hsu H-C (2020). Effects of a cultural competence education programme on clinical nurses: a randomised controlled trial. Nurse Educ Today.

[46] Zarzycka D, Chrzan-Rodak A, Bąk J, Niedorys-Karczmarczyk B, Ślusarska B (2020). Nurse cultural competence-cultural adaptation and validation of the polish version of the nurse cultural competence scale and preliminary research results. PLoS One.

[47] Thackrah RD, Wood J, Thompson SC (2020). Cultural respect in midwifery service provision for Aboriginal women: longitudinal follow-up reveals the enduring legacy of targeted program initiatives. Int J Equity Health.

[48] Ozkara San E (2019). Effect of the diverse standardized patient simulation (DSPS) cultural competence education strategy on nursing students’ transcultural self-efficacy perceptions. J Transcult Nurs.

[49] Daugherty HN, Kearney RC (2017). Measuring the impact of cultural competence training for dental hygiene students. Am Dent Hyg Assoc.

[50] Singleton JK (2017). An enhanced cultural competence curriculum and changes in transcultural self-efficacy in doctor of nursing practice students. J Transcult Nurs.

[51] Saffee CL (2015). Improving the cultural competency of registered nurses in a Bachlor’s completion program. J Transcult Nurs.

[52] Birgili B. Comparative reflection on best known instructional design models: notes from the field. Curr Issues Emerg eLearning. 2019;6(1):78–94.

[53] Allen M: Designing online asynchronous information literacy instruction using the ADDIE model. In: Distributed Learning edn. Elsevier; 2017. p. 69–91.

[54] Gawlik-Kobylinska M (2018). Reconciling ADDIE and agile instructional design models—case study. New Trends Issues Proc Humanit Soc Sci.

